# A clinical study on minimally invasive percutaneous nephrolithotomy combined with ureteral access sheath for the treatment of kidney stones

**DOI:** 10.3389/fsurg.2025.1557603

**Published:** 2025-07-29

**Authors:** Boyang Liu, Yanjie Kang, Yafeng Shang

**Affiliations:** ^1^ Department of Urology, Xinxiang Medical University, Xinxiang, China; ^2^ Department of Urology, Luoyang Central Hospital Affiliated to Zhengzhou University, Luoyang, China

**Keywords:** MPCNL, kidney stones (KSs), ureteral access sheath, efficacy, safety

## Abstract

**Objective:**

To investigate the efficacy and safety of minimally invasive percutaneous nephrolithotomy (MPCNL) combined with ureteral access sheath in the treatment of complex kidney stones.

**Methods:**

Seventy patients with complex kidney stones in the author's hospital from June 2022 to December 2023 were randomized. There were 35 cases of minimally invasive percutaneous nephrolithotomy combined with ureteral access sheath and 35 cases of minimally invasive percutaneous nephrolithotomy alone.

**Results:**

Compared to the MPCNL group, the MPCNL + UAS group demonstrated a significantly shorter operative time (55.4 ± 9.2 min vs. 61.5 ± 12.8 min, *p* = 0.027), significantly lower intraoperative renal pelvic pressure (9.15 ± 4.13 mmHg vs. 11.35 ± 4.21 mmHg, *p* = 0.031), and a significantly lower incidence of postoperative Clavien complications (*p* < 0.05); however, there were no significant differences between the groups in postoperative creatinine change, stone clearance rate at 1 day postoperatively, or stone clearance rate at 30 days postoperatively (*p* > 0.05).

**Conclusions:**

Minimally invasive percutaneous nephrolithotomy combined with ureteral access sheath is safe and effective in the treatment of complex kidney stones. Ureteral access sheath can significantly shorten the operation time of minimally invasive PCNL, keep the visual field clear, reduce the pressure of renal pelvis, and reduce the incidence of complications.

## Introduction

Complex kidney stones represent a significant focus in stone management, with standard percutaneous nephrolithotomy (PCNL) serving as the mainstream surgical approach (1). However, due to the substantial trauma associated with its larger tracts, urologists increasingly utilize miniaturized PCNL techniques such as mini-PCNL, ultra-mini-PCNL, and super-mini-PCNL (2, 3). Clinically, we observed that during minimally invasive PCNL, fragments frequently migrated and accumulated toward the ureter, forming ’stone streets’. This led to increased difficulty in fragment retrieval, prolonged operative time, and necessitated repositioning for ureteroscopic lithotripsy in some cases. To address this limitation, we implemented a combined approach using a ureteral access sheath (UAS) for complex kidney stones.

## Materials and methods

### Research design

This retrospective study evaluated 70 patients with complex renal stones admitted to the authors’ hospital between June 2022 and December 2023. The patients were divided into two groups based on the surgical method selected: MPCNL (*n* = 35) and MPCNL + UAS (*n* = 35).

Inclusion Criteria:

1. Age 18–75 years old.
2. First-time treatment for kidney stones.

Exclusion Criteria:

1. Ectopic kidney or solitary kidney stones;
2. Complicated with ureteral stones, renal tuberculosis, renal tumors, renal insufficiency, acute or chronic nephritis, or nephrotic syndrome;
3. Pregnant women;
4. Patients with severe heart, liver, or hematological system diseases;
5. Severe spinal deformity.

All patients underwent comprehensive medical history collection and physical examination. Preoperative assessment of the urinary system was performed using intravenous pyelography (IVP) and non-contrast CT. Stone size was determined by the maximum diameter of the stone. Patients with positive preoperative urine cultures received sensitive antibiotic therapy until urine culture results turned negative. All patients underwent x-ray (KUB) and ultrasound examinations on postoperative day 1. Residual stones <4 mm were defined as stone-free status. If patients achieved stone-free status without significant bleeding or infection, the nephrostomy tube was removed, the urinary catheter was removed within 48 h, and the double-J stent was removed via cystoscopy 2 weeks postoperatively. Follow-up at 30 days postoperatively was conducted using non-contrast CT. Complications for all patients were recorded according to the modified Clavien complication classification system.

### Surgical methods

MPCNL combined with USA group: Under general anesthesia, the patient was placed in the oblique supine running position, with the affected side upward. The height of the operating table on both sides was adjusted to be suitable for the operator's operation. The rigid ureteroscope was inserted into the ureter on the affected side, and a nitinol guide wire was retained to the renal pelvis. A 12/14F ureteral access sheath (UAS) was placed along the guide wire. Artificial hydronephrosis was established by perfusing normal saline through the obturator of the ureteral access sheath. Under ultrasound guidance, 18G puncture needle was inserted into the target calyceum, the fascial dilator was expanded and placed into the 18F outer sheath, and the UAS obturator was pulled out. wolf nephroscope and 550 μm holmium laser fiber lithotripsy were used. The removal of stones depends on pressure flushing, UAS discharging and grasping. After confirming the removal of the calculi, the ureteral passage sheath was removed, and retrograde indwelling double J tubes, 16F nephrostomy tubes and urinary tubes were placed. The ureteral catheter with head end opening was placed into the renal pelvis via UAS, one end was connected to the IBP channel of the anesthesia monitor, and the pressure sensor was used for real-time measurement.

MPCNL group: Under general anesthesia, lithotomy position, two 5F ureteral catheters were inserted into the affected ureter under ureteroscopy, placed into the renal pelvis, and 16F three-cavity ureteral catheter was indwelled in the bladder and fixed with the ureteral catheter. Then the lateral position was changed, and artificial hydronephrosis was established. The channel was perforated through the calyceal of the posterior group under the guidance of ultrasound, and the channel was expanded to 18F under the guidance of guide wire. Wolf nephroscope was implanted and lithotripsy was performed with 550 μm holmium laser fiber. The ureteral catheter with head end opening was placed into the renal pelvis via UAS, one end was connected to the IBP channel of the anesthesia monitor, and the pressure sensor was used for real-time measurement.

### Statistical analysis

SPSS 22.0 was used for data analysis. Presented as mean ± standard deviation, were analyzed using *t*-tests. Counting data, presented as percentages, were analyzed using chi-square (*χ*^2^) tests. A *P*-value of less than 0.05 was considered statistically significant.

### Ethical approval

This study was conducted retrospectively, utilizing anonymous data that had been previously collected during patient assessments or for service evaluation. The local ethics committee reviewed the study proposal and determined that ethical approval was not required.

## Results

As shown in Table 1, preoperative data of the two groups, such as age, gender, left and right side, body mass index (BMI), hydronephrosis, stone size, and CT value of stones, showed no statistical difference and were comparable. The average stone size of MPCNL group was 4.0 ± 0.8 cm, including 3 complete antler stones, 2 partial antler stones. The average stone size of MPCNL combined with UAS group was 4.1 ± 0.7 cm, including 2 complete antler stones, 5 partial antler stones.

**Table 1:** Patient characteristics at baseline.

Variable	MPCNL (*n* = 35)	MPCNL + UAS (*n* = 35)	*P*-value
Age	48.7 ± 12.5	46.4 ± 9.4	0.380
Male/Female	13/22	10/25	0.445
Right/left	20/15	26/9	0.131
BMI(Kg/m^2^)	24.4 ± 2.8	25.6 ± 2.9	0.091
Degree of hydronephrosis: no hydronephrosis or mild hydronephrosis	19	25	0.138
Degree of hydronephrosis: moderate and severe hydronephrosis	16	10	0.139

* *P* > 0.05 is considered statistically non-significant.

As shown in Table 2, patients in the MPCNL combined with USA group had significantly lower mean operation time, intraoperative renal pelvis pressure and incidence of complications than those in the MPCNL group, *P* < 0.05 was considered statistically significant. There was no significant difference in stone free rate (SFR) between the two groups at 1 day and 30 days after operation. There was no significant difference between the two groups in the decrease of hemoglobin and the change of creatinine before and after operation.

**Table 2:** Comparison of postoperative data between the two groups.

Variable		MPCNL (*n* = 35)	MPCNL + UAS (*n* = 35)	*P*-value
Mean operation time (min)		61.5 ± 12.8	55.4 ± 9.2	0.027
Decreased hemoglobin (g/L)		10.1 ± 7.6	12.9 ± 5.8	0.090
Creatinine change (μmol/L)		11.2 ± 6.0	9.2 ± 4.4	0.131
Stone clearance rate (1 day after surgery)		25 (71.4%)	31 (88.5%)	0.073
Stone clearance rate (30 days after surgery)		30 (85.7%)	34 (97.1%)	0.088
Intraoperative pelvic pressure (mmHg)		11.35 ± 4.21	9.15 ± 4.13	0.031
Clavien complications		10 (28.5%)	4 (11.4%)	0.031
Class I	Fever (>38.5℃)	7	2	
	Pain	1	2	
Class II		1	0	
Class III		1	0	

* *P* < 0.05 is considered statistically significant.

## Discussion

Complex kidney stones, defined as those larger than 2.5 cm in diameter, multiple renal calculi, or staghorn calculi, present significant clinical challenges (4). Key treatment goals include minimizing renal injury, preventing urosepsis, and achieving complete stone clearance. Although standard PCNL offers high stone-free rates (SFR), its larger percutaneous tracts are associated with greater renal trauma and higher complication rates. A multicenter study of 5,803 patients across 96 centers reported a 14.5% postoperative complication rate for standard PCNL, including severe bleeding events (5). Recently, innovative technologies like the flexible and navigable suction ureteral access sheath (FANS) and digital imaging and surgical system (DISS) have emerged as optimized solutions for complex stones. The FANS system utilizes unique design features to enhance fragment clearance efficiency and stabilize the surgical field. Integrated with negative pressure suction, it significantly reduces operative time, improves SFR, and lowers postoperative complication rates. Complementarily, the DISS system employs advanced digital imaging to provide superior visual clarity and precise instrument control, enhancing procedural safety and efficacy. These systems demonstrate substantial clinical advantages, expanding therapeutic options for complex renal stone management.

To mitigate complications linked to standard PCNL access, efforts have focused on minimizing tract diameter. However, smaller tracts often prolong operative time. Micro-perc tracts (typically F14-F20, with F16-F20 being most prevalent) utilize lithotripsy modalities including holmium laser, pneumatic devices, or EMS systems. In settings lacking EMS, holmium laser or pneumatic lithotripsy predominates, with fragment evacuation relying primarily on pressure irrigation and basket retrieval. Elevated intrarenal pressure during irrigation frequently causes fragments to migrate toward lower-pressure regions such as the ureteropelvic junction or ureter. This complicates fragment removal, necessitating nephroscope exchange for ureteroscopic retrieval (antegrade or retrograde). Clinically, we observed that preoperative dual ureteral catheter placement failed to prevent such migration. Notably, recent studies report that thulium fiber laser-assisted MPCNL achieves superior stone pulverization (“dusting”), effectively minimizing stone street formation (6). Nevertheless, further clinical validation is required to establish the definitive role of thulium laser in urinary calculi management.

Incorporating a ureteral access sheath (UAS) enables efficient fragment evacuation through the lumen via irrigation flow, preventing ureteral stone street formation and eliminating repeated nephroscope reinsertion for fragment retrieval. Concurrently, the UAS physically obstructs larger fragment migration down the ureter, further reducing operative time. Moreover, UAS enhances visual clarity during procedures. Renal hemorrhage and holmium laser-induced vaporization “mist” can obscure the surgical field, often necessitating procedural pauses during significant bleeding. By lowering intrarenal pressure, UAS permits higher irrigation flow rates, accelerating fluid clearance. The larger UAS lumen improves drainage efficiency, preventing intrarenal blood clot accumulation and thereby maintaining a clear surgical view—a critical factor enabling shorter operative durations. Notably, mPCNL with UAS increases procedural costs by approximately ¥4,000 (RMB).

Although miniaturized tracts reduce renal injury, maintaining adequate visualization requires increased irrigation flow rates. However, restricted outflow through narrow sheaths elevates intrarenal pressure (7). Additionally, rapid nephroscope deflection, calyceal neck positioning, instrument insertion for fragment retrieval, or sheath obstruction by calculi may cause abrupt intrapelvic pressure spikes (8). Hese pressure increases impair renal function and heighten risks of infection and fluid absorption. UAS effectively lowers intrapelvic pressure, decreases irrigation fluid absorption, and reduces infectious complications. Urosepsis remains among the most severe PCNL complications (9), with reflux fever and systemic fluid absorption being frequent adverse events (10, 11). Reported incidences of PCNL-related urosepsis and septic shock range from 0.3% to 4.7%, with mortality rates of 25%–60% (12). Beyond bacteremia from calculi, elevated irrigation pressure or prolonged high-pressure exposure during lithotripsy promotes pyelovenous/pyelolymphatic reflux. This facilitates bacterial/endotoxin translocation into systemic circulation, increasing sepsis risk (13). Hong et al. monitored renal pelvic pressure via percutaneous tracts during MPCNL, demonstrating that cumulative duration >30 mmHg correlates with reflux fever risk (10). Dogan et al. documented postoperative fever in 21% (17/81) of cases, directly linked to fluid absorption (11). Systemic fluid absorption during PCNL occurs through three pathways: pyelolymphatic reflux, pyelovenous backflow, and fornical rupture (8), increasing cardiac preload. Consequently, patients with cardiac impairment require heightened vigilance.Studies confirm that even when maintaining pressure <30 mmHg, larger tracts (20F/22F) achieve lower intrarenal pressures than smaller micro-perc sheaths (16F/18F), corresponding to reduced postoperative infection rates (14). Thus, minimizing operative time and intrapelvic pressure is critical for preventing infectious complications and fluid overload. The 12/14F UAS accommodates fragments <3.8–4.4 mm, accelerating clearance while reducing pressure. This study observed sustained intrarenal pressures of 5–15 mmHg with UAS despite high-flow irrigation. Lower pressure and shorter duration collectively decrease fluid extravasation/absorption, reducing complication incidence.

UAS application demonstrates a favorable safety profile when deployed with atraumatic technique (15), Ureteral blood flow studies reveal transient ischemia during UAS placement: 12F–14F UAS reduces flow to <50% of baseline, 14F–16F UAS reduces flow to <50% of baseline, 10–12F UAS reduces flow by 25% from baseline. This ischemia rarely progresses to necrosis due to compensatory mechanisms in the ureteral wall that rapidly restore near-baseline perfusion, preserving urothelial integrity (16). A prospective analysis of 72 UAS-assisted procedures found no association with postoperative ureteral stricture (17). UAS-related injuries are predominantly low-grade (Clavien 0-I, 76.8%), with higher-grade injuries primarily classified as grade II (20.5%). These are typically self-limiting without sequelae. Crucially, no grade IV injuries requiring open repair have been documented (18).

Furthermore, compared to traditional PCNL, this combined approach eliminates intraoperative repositioning from lithotomy to prone/lateral positions, significantly streamlining patient setup. Fragment clearance via irrigation proves markedly easier in the oblique supine lithotomy position than in full lateral or prone positions.Should significant residual stones remain inaccessible percutaneously, concurrent retrograde intrarenal surgery (RIRS) can be performed. Endoscopic Combined Intrarenal Surgery (ECIRS) — integrating antegrade and retrograde access — enables direct visual guidance during puncture and lithotripsy. This reduces renal parenchymal injury and bleeding risks while lowering intrarenal pressure, consequently diminishing postoperative infection rates. Critically, ECIRS demonstrates distinct advantages for complex scenarios (e.g., multiple calculi, staghorn stones), achieving superior stone-free rates with fewer complications.

Our study has several limitations. This study has limitations that warrant acknowledgment: First, its retrospective nature limited control over treatment allocation. Senior surgeons selected UAS application based on perceived stone complexity (e.g., staghorn calculus burden, pelvicalyceal anatomy) and estimated bleeding risk, potentially introducing selection bias as the UAS group may represent patients more amenable to benefit. Second, the modest cohort size (*n* = 70; 35 per group) lacked prospective power calculation. Larger studies are needed to validate observed differences—particularly the 11.4% disparity in 30-day stone-free rates—for clinical significance.Furthermore, as conventional MPCNL dominates complex stone management in China and UAS-assisted procedures are institutionally restricted, accruing larger cohorts remains challenging.

## Conclusions

Ureteral access sheath can significantly shorten the operation time of minimally invasive PCNL, keep the visual field clear, reduce the pressure of renal pelvis, reduce the incidence of complications, and does not increase the risk of ureteral injury, but it seems to have no significant effect on the improvement of stone clearance rate.

## Data Availability

The original contributions presented in the study are included in the article/Supplementary Material, further inquiries can be directed to the corresponding author.
